# Point-of-care ultrasound (POCUS) in the surgery of a paraplegic pregnant female: A case of “do no harm”

**DOI:** 10.1016/j.radcr.2024.04.087

**Published:** 2024-06-18

**Authors:** Mobin Ibne Mokbul, Shahnawas Biswas, Shreya Singh Beniwal, Srishti Sharma, Kareema Rebekah Sofia Cummings, Robiul Karim, Md. Sumon Rana

**Affiliations:** a Dhaka Medical College Hospital, Dhaka, Bangladesh; b Department of Neurosurgery, BRB Hospital, Dhaka, Bangladesh; c Lady Hardinge Medical College, New Delhi, India; d Saraswati Medical College, Unnao, Uttar Pradesh, India; e College of Medical Sciences, University of Guyana, Turkeyen Campus, Greater Georgetown, Guyana

**Keywords:** Point-of-care ultrasound (POCUS), Pregnancy, Paraplegia, Spinal tumor, Surgery, Spine

## Abstract

We present here a case of multidisciplinary management of a 20-year-old pregnant woman who presented with sudden paraplegia attributed to a large paraspinal tumor. Magnetic resonance imaging (MRI) revealed compressive dorsal myelopathy due to an extramedullary tumor. Given the urgency of her symptoms and pregnancy status, a multidisciplinary team decided to proceed with surgery while avoiding radiation exposure (eg, O/C-arm). Intraoperative point-of-care ultrasound (POCUS) was utilized for tumor localization and surgical guidance, facilitating successful gross total excision with minimal risk to the fetus. Postoperative recovery was uneventful, with improvement in muscle strength and preservation of the pregnancy. Beyond tumor localization, POCUS offers additional benefits in assessing maternal hemodynamics and detecting potential complications. This case highlights the utility of POCUS as a radiation-free theranostic imaging modality in pregnant patients with spinal tumors, enhancing safety in surgery and optimizing outcomes for both mother and fetus.

## Case description

**Fig. 1:**
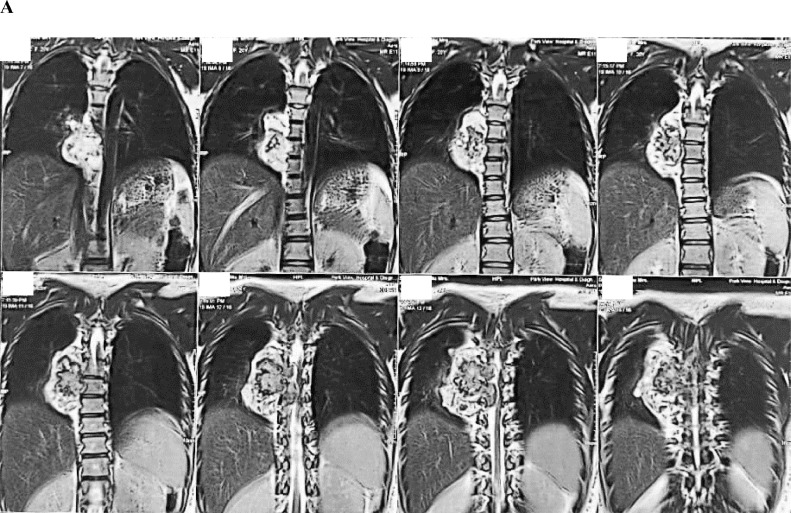

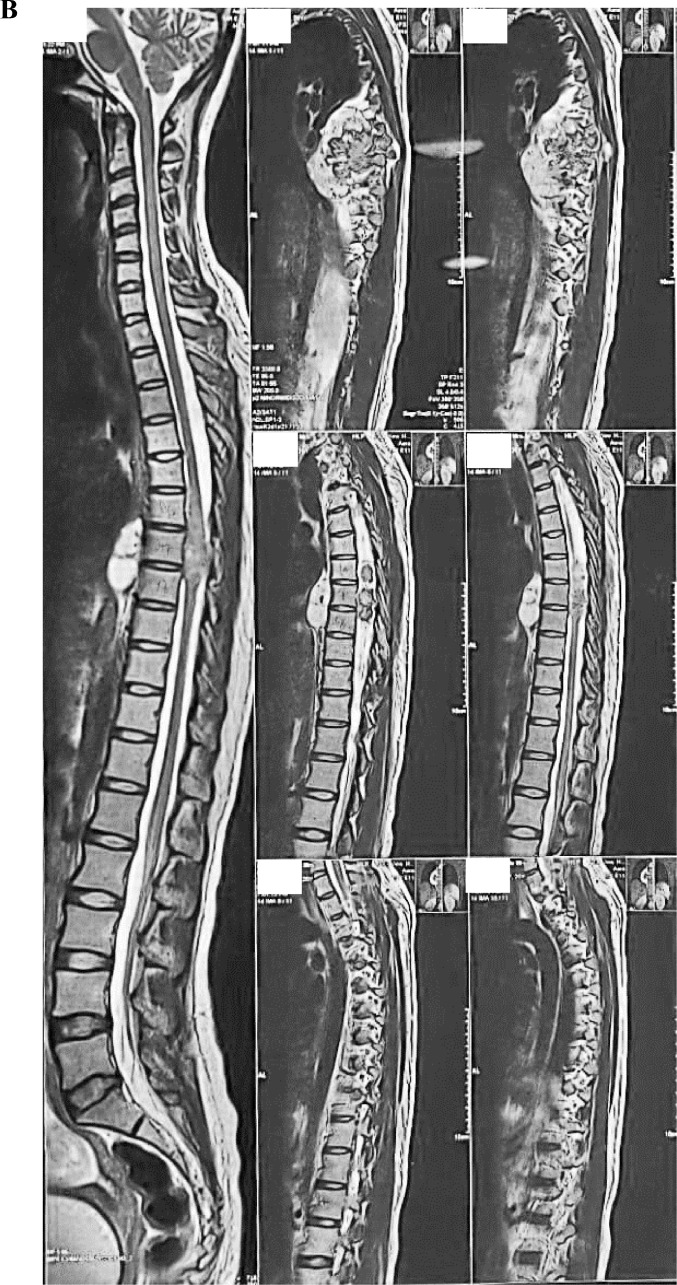

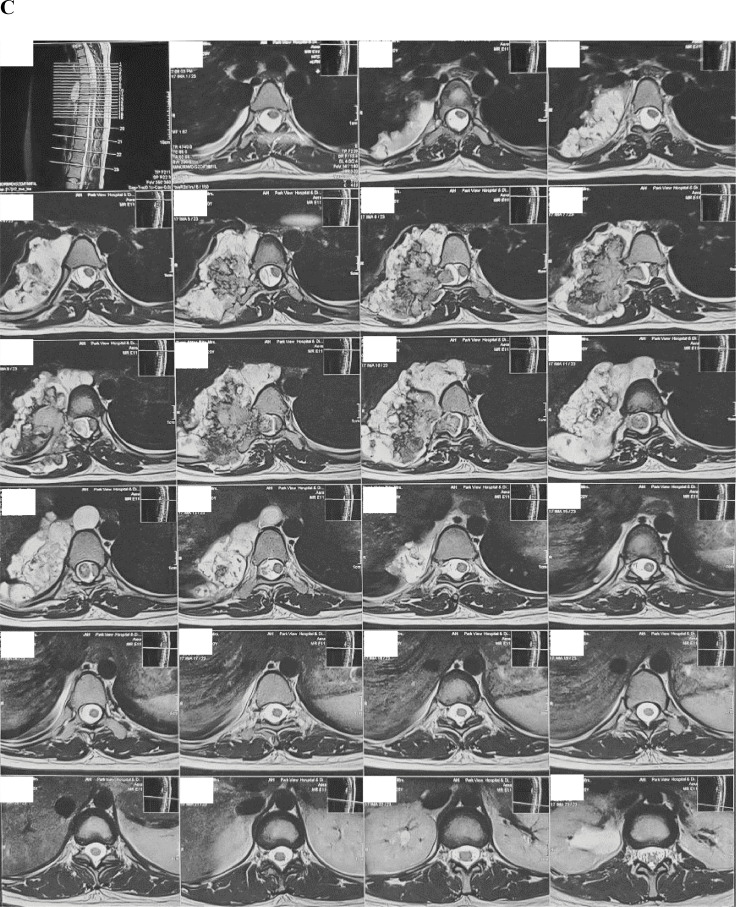
MRI images show a large right paraspinal tumor at level T_6_-T_9_ compressing the spinal cord involving the right parietal pleura. (A) Coronal plane MRI: T2-weighted sequence without gadolinium enhancement, (B) Sagittal plane MRI: T2-weighted sequence without gadolinium enhancement and (C) Axial plane MRI: T2-weighted sequence without gadolinium enhancement.

A 20-year-old woman presented in her first trimester with sudden onset paraplegia. On further history, her limb weakness has aggravated for last 2 months initially having paraparesis. She also denied any history of meningitis, tuberculosis, metabolic disorders, diarrhea, or any central nervous system infections in the past. Magnetic resonance imaging (MRI) revealed compressive dorsal myelopathy due to a large right paraspinal (extramedullary) tumor at level T_6_-T_9_ compressing the spinal cord and also involving the right parietal pleura (Fig. 1). Initially, the short onset of symptoms and its surrounding extension to the pleura was concerning for the surgical team as a malignancy.

Though there is no standard established management guidelines exist for spinal tumors during pregnancy, literature suggest spinal surgery in first trimester only if there is progressive neurologic deterioration [1]. Due to our patient's rapid deterioration of symptoms, multidisciplinary consisting of neurosurgeon, obstetrician, and anesthesiologist decided to operate on the patient while avoiding radiation exposure and other teratogens to ensure maximum outcome for both the mother and fetus (performed in 16 weeks of pregnancy). Proper surgical positioning (eg, prone position) during surgery was done in the operating table with an abdominal binder.

**Fig. 2:**
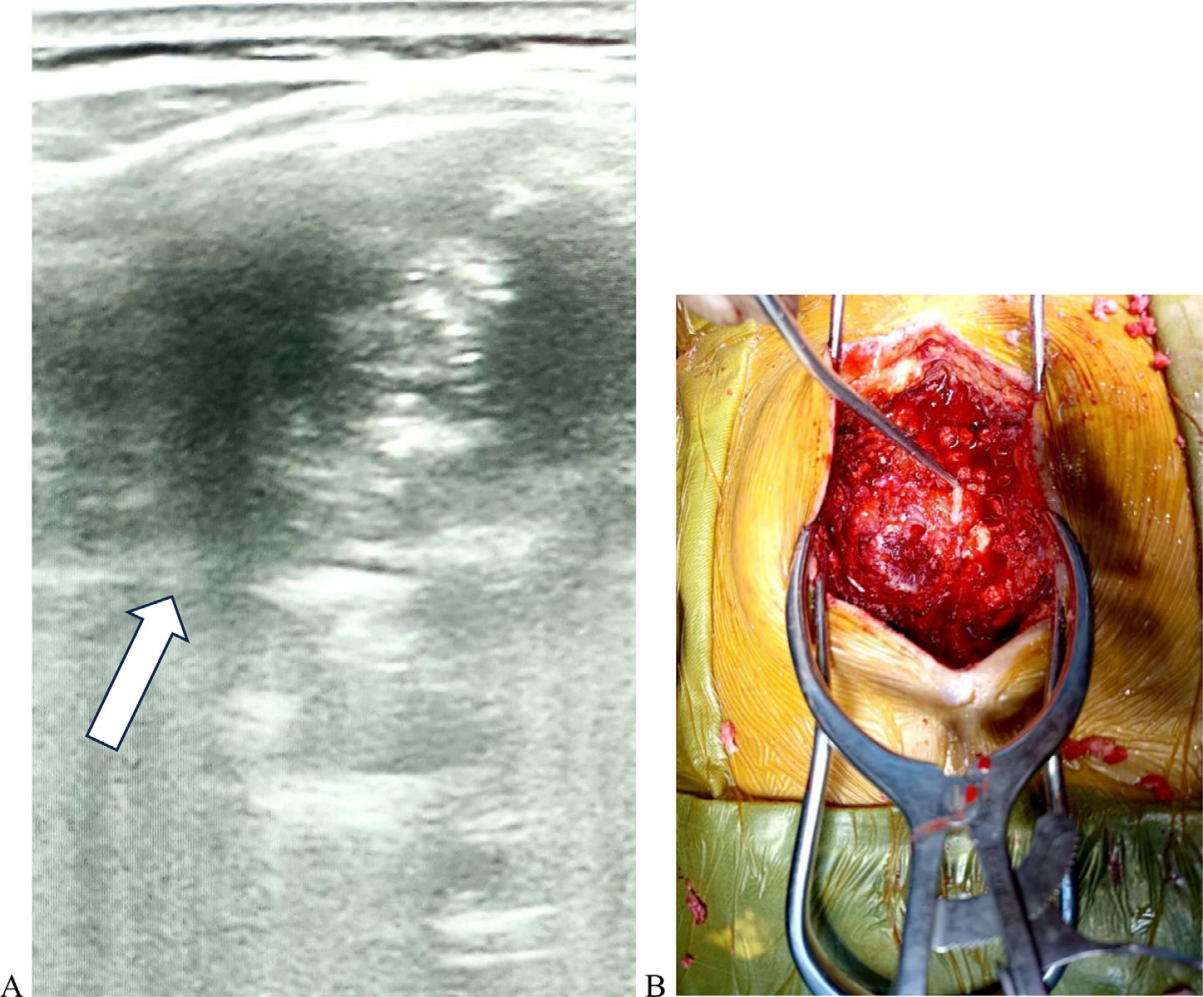
(A) Intraoperative POCUS to guide the tumor (marked in white arrow shows the tumor as hypoechoic lesion) (B) Guiding surgical site through POCUS.

**Fig. 3:**
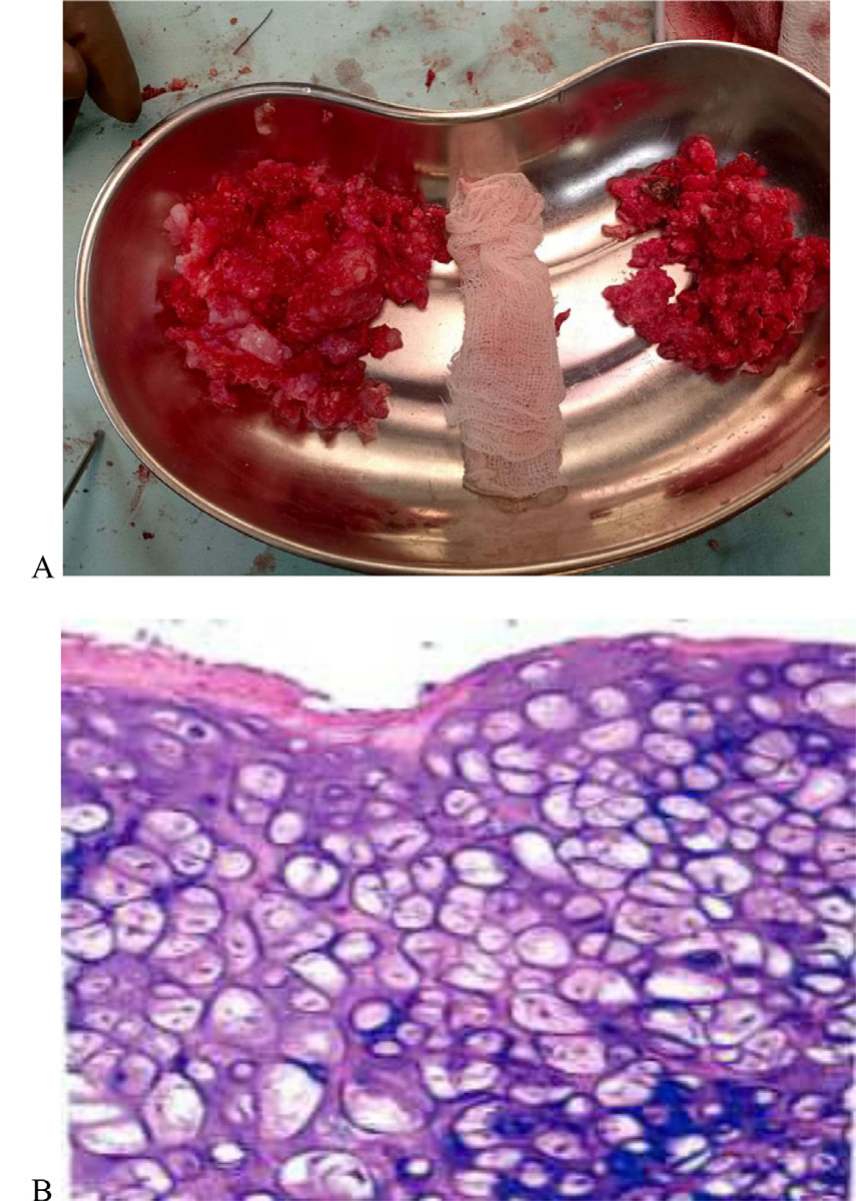
(A) Resected sample following surgery. (B) Histopathology showing hyaline cartilage encompassed by bone.

During the spinal surgery, we avoided traditional O-arm/C-arm to avoid radiation exposure and used POCUS to locate the lesion (Fig. 2). Utilizing POCUS, precise tumor localization was achieved, guiding surgical incision site. We were able to achieve gross total excision of the lesion by laminectomy and bilateral fixation on the right side between T_6_-T_9_ discs with a neurosurgical microscope. Frozen section biopsy during surgery and histopathology of resected sample from lung and spine after surgery revealed an osteochondroma (Fig. 3). No granulomatous, dysplastic, or malignant changes were seen. The anatomic origin of the tumor was thought to be cartilages arising from the vertebra. Drainage tubes on spine and chest were placed. She was released from the neurosurgery department 1 week after the surgery and was close observation under the obstetric team. Her muscle physical power improved from Grade 1 to Grade 4 during release in Medical Research Council (MRC) scale for muscle strength. Her pregnancy status and fetus were completely fine up to 18 weeks of pregnancy as monitored by obstetrics department till the period she was admitted to our hospital. Further obstetric information was not available as she did not come for follow-up and was hailing from a distant district of the country.

## Discussion

POCUS was instrumental throughout the surgical procedure of our patient. Intraoperatively, POCUS provided real-time imaging, enabling accurate localization of the tumor without the use of radiation. This allowed for precise surgical planning and execution, ensuring complete tumor excision while minimizing potential harm to the fetus. POCUS can also facilitate monitoring of fetal well-being intraoperatively, providing valuable information to the obstetric team. Postoperatively, POCUS can also be utilized to confirm the success of tumor resection and assess for any complications, offering a comprehensive evaluation without the need for additional radiation exposure.

Prone and lateral tilt position are positions of choice in pregnant women with any dorsal pathology [2]. About >22 weeks of gestation, fetal heart monitoring should be used during anesthesia [3]. During pregnancy, due to surge of Brain-derived neurotrophic factor (BDNF), CNS benign tumors may mimic malignancy due to increased proliferation rate [4]. If needed, exposure of radiation in the first trimester is associated with an increased risk of major birth defects, whereas use in the second and third trimesters is associated with intrauterine growth restriction, low birthweight and stillbirth [5], [6].

In addition to its utility in guiding surgical interventions, the use of point-of-care ultrasound (POCUS) in pregnant patients offers several advantages beyond its role in tumor localization. POCUS serves as a non-invasive imaging modality that can aid in the assessment of maternal hemodynamics, fluid status, and cardiac function, all of which are critical considerations during surgery in pregnant individuals. Furthermore, POCUS enables rapid and accurate diagnosis of potential complications such as deep vein thrombosis and pulmonary embolism, which may arise due to the hypercoagulable state of pregnancy and prolonged immobilization during surgery. By facilitating prompt detection and intervention for such complications, POCUS contributes to improved maternal outcomes and ensures the safety of both the mother and fetus throughout the perioperative period. Moreover, its portability and real-time imaging capabilities make POCUS a valuable tool for obstetricians, anesthesiologists, and surgeons alike, enhancing interdisciplinary collaboration and optimizing patient care in complex clinical scenarios.

## Conclusion

In pregnant patients with spinal tumors, minimizing radiation exposure is paramount. This case underscores the utility of POCUS as a safe and effective imaging modality in such scenarios. By providing real-time imaging without radiation, POCUS enables precise localization and guidance during surgical procedures while ensuring optimal outcomes for both mother and fetus. As demonstrated in this case, POCUS emerges as a valuable theranostic tool in the multidisciplinary management of spinal tumors during pregnancy, offering a radiation-free alternative for diganostic, therapeutic, and surgical interventions.

## Patient consent

Necessary consents have been taken by the senior author.

## References

[1] Fujii K, Orisaka M, Yamamoto M, Nishijima K, Yoshida Y. (2018). Primary intramedullary spinal cord tumour in pregnancy: a case report. Spinal Cord Ser Cases.

[2] Bongetta D, Versace A, De Pirro A, Gemma M, Bernardo L, Cetin I (2020). Positioning issues of spinal surgery during pregnancy. World Neurosurg.

[3] Po' G, Olivieri C, Rose CH, Saccone G, McCurdy R, Berghella V (2019). Intraoperative fetal heart monitoring for non-obstetric surgery: a systematic review. Eur J Obstet Gynecol Reprod Biol.

[4] Yin C, Qi X. (2017). Pregnancy promotes pituitary tumors by increasing the rate of the cell cycle. Oncol Lett.

[5] Ngu SF, Ngan HY. (2016). Chemotherapy in pregnancy. Best Pract Res Clin Obstet Gynaecol.

[6] Sakowicz A, Dalton S, McPherson JA, Charles AG, Stamilio DM. (2023). Accuracy and utilization patterns of intraabdominal imaging for major trauma in pregnancy. Am J Obstet Gynecol MFM.

